# ‘The Husband, For Whom She Endures All This’: Dutch Men in Childbirth, 1900–1940

**DOI:** 10.1093/shm/hkad088

**Published:** 2024-01-13

**Authors:** Hieke Huistra

**Affiliations:** Freudenthal Institute/Descartes Centre, Utrecht University, Utrecht, the Netherlands

**Keywords:** childbirth, fathers, husbands, Netherlands, emotions

## Abstract

I argue that in the early twentieth-century Netherlands, fathers regularly attended the birth of their children, and that this attendance was generally accepted or even encouraged by doctors. My findings contrast with existing historiography on the Anglo-Saxon countries, where, at the time, fathers were usually not present at births. I explain this difference between the Netherlands and the Anglo-Saxon countries through the ideal of the harmonious family that permeated Dutch society at the time. I show how birth was seen as a family event, in which the father should be emotionally involved. Men had to manage this emotional involvement carefully: they had to display emotions without losing control of these emotions. My findings show that we need to study doctor-led births in order to fully understand the slow rise of hospital births in the Netherlands.

It happened during fair time, just over a century ago. In the city of Utrecht, the Netherlands, an expectant woman had gone into labour. Someone went to the pub to fetch her husband, so that he could attend the birth. Unfortunately, the husband was both drunk and in possession of an accordion. Each time his wife experienced a contraction, he played the same tune on the accordion over and over, declaring that he just could not stand ‘the moaning’. When the contraction had passed, the man would fall back into ‘lethargic indifference’, according to obstetrician Maurits Muller, who tells this story in his memoirs. All in all, the other attendants (a trainee doctor, a maternity nurse and one or two neighbours) considered the husband’s presence so unhelpful that they wanted him out. Since they did not succeed in removing him, they called in a more senior obstetrician to help, most likely Muller. Muller talked and talked, but failed; the husband insisted that ‘my place is beside my wife and I am not leaving’.^1^ A policeman was asked to stand in front of the window, as a threat, but this also did not have the desired effect. Ultimately, what worked was using the carrot instead of the stick: the trainee doctor put something in the man’s hands and whispered something in his ear, after which the husband’s face lit up, and he left—no doubt on his way to the pub, to have another drink and play another tune.

This baby came into the world without the father present. But the story told about his or her birth reveals that many other Dutch children entered the world with their fathers there to welcome them. First, note that the father was fetched from the pub to attend the birth. He did not just happen to be at home when his wife went into labour; he was expressly collected to allow him to attend. Furthermore, drunk as he was, his remark that ‘his place was beside his wife’ seems to appeal to some cultural notion about masculine behaviour that allowed and even encouraged husbands to attend their wives’ labours.

This article aims to flesh out that notion. It investigates the role Dutch fathers played in childbirth between roughly 1900 and 1940, through analysing birth stories in doctors’ memoirs and parents’ diaries, and arguments against hospital births in medical journals. Based on these sources, I argue that Dutch fathers of all social classes regularly attended childbirth and that medical professionals usually accepted their presence. I suggest that this practice was shaped by the notion of birth as a family event to be experienced by both spouses together, which fit the Dutch ideal of the harmonious family. To properly share the experience, husbands had to participate in the birth *emotionally*. I argue that they had to manage this emotional involvement carefully: they had to display emotions but simultaneously remain in control of these emotions.

The widespread presence of fathers in Dutch birthing rooms contrasts with existing historiography. Until now, most work on fathers in childbirth has focused on the Anglo-Saxon countries, particularly the United States and the British Isles.^2^ In 1981, Jill Suitor as one of the first historians addressed the role of men in childbirth. She showed that in the nineteenth-century United States, husbands frequently accompanied their wives during birth.^3^ A few years later, Patricia Jalland and Judith Lewis showed the same for nineteenth-century Britain, at least for the higher classes.^4^ Leanne Calvert has more recently presented an early nineteenth-century Irish case study also revealing husbands’ involvement in childbirth.^5^ But by the 1910s, husbands had disappeared from the birthing room in both Britain and the United States, according to the work of Laura King and Judith Leavitt—and they would not return until the 1950s and 1960s.^6^ Yet, Dutch husbands were very much present in the birthing room in the first half of the twentieth century, and doctors were happy to have them. Thus, my research on the Netherlands shows that the Anglo-Saxon picture cannot be simply generalised to other countries. I focus on the period between 1900 and 1940 because this is when the difference is most striking. I argue that this difference can be explained through the ideal of the harmonious family that permeated Dutch society at the time.

In doing so, I focus on doctor-led births. This is a consequence of my sources—more on that below—but this focus also lets me complicate existing historiography on the transition from home to hospital birth, or lack thereof. In the Netherlands, the number of hospital births rose slower than elsewhere. In the early 1950s, just over 20 per cent of Dutch births were hospital births.^7^ By then, almost 60 per cent of UK births and 90 per cent of US births took place in the hospital.^8^ At the end of the twentieth century, almost one in three Dutch women still gave birth at home.^9^ This difference became first visible in the period discussed in this article.

Sociologists, historians and medical professionals often point towards the strong position of the Dutch midwife to explain the relatively high number of home births in the Netherlands.^10^ Yet, midwives were not the only player in the Dutch birthing system: doctors supervised a significant share of births. ‘Doctors’ here includes both general practitioners and specialised obstetricians. In the 1910s, doctors supervised almost 40 per cent of all births.^11^ Midwives supervised almost 60 per cent, and around 3 per cent of the births happened without any medical professional present. By the end of the 1930s, the midwives’ share had decreased to less than half of all births. By then, most births—just over 50 per cent—were doctor-led, and just 1 per cent of births were without professional supervision. In the 1940s, the doctors’ share would increase further to about 60 per cent. Most supervising doctors were general physicians; obstetricians formed a small minority.^12^ In both public and medical debates, however, specialised obstetricians were significant voices. Only obstetricians could supervise hospital births, but most births took place at home: in 1900, only about 2 per cent were hospital births; in 1940, this had grown to around 13 per cent.^13^ Home births could be supervised by midwives, general physicians and obstetricians alike. The doctors could supervise every birth they wanted to, but midwives were only allowed to supervise so-called ‘natural’ births; as soon as complications arose, a doctor had to take over.

Yet, although doctors supervised around 40–60 per cent of all births in the first half of the twentieth century, doctor-led births have seldom been considered by historians and sociologists studying the Dutch birthing system. My research partly fills this gap. It shows how doctor-led births could be family events, taking place at home, and that doctors, even obstetricians, explicitly advocated for this type of birth. Thus, my research reveals that we need to look beyond the midwife to understand the high number of home births in the Netherlands.

The article is structured as follows. First, I discuss my source selection and the advantages and limitations of my sources. Next, I explain the Dutch ideal of the harmonious family, which I subsequently connect to doctors’ ideas on birth as a family event. Then, I analyse doctors’ and parents’ birth descriptions to show how fathers had to simultaneously display and control their emotions. Throughout the article, I provide examples that substantiate my claim that fathers regularly attended the birth of their children.

Before we move on to the sources, some words on terminology. My historical actors use both ‘father’ and ‘husband’ to describe the men attending childbirth. I follow their usage, meaning that throughout the article, I will use both words. Some historians have found that in the nineteenth century, men gained access to the birthing room as husbands, not as fathers.^14^ In studying my sources, I have not become convinced that this also applied to the early twentieth-century Netherlands.

## Sources

I have used three main types of sources. First, doctors’ published memoirs; second, parents’ unpublished diaries and recollections and third, medical periodicals. Most historians studying men in childbirth relied heavily on first-person birth descriptions: Lewis and Jalland scrutinised letters and diaries; King used oral history interviews; Leavitt worked with a magnificent collection of hospital waiting room ‘fathers’ books’.^15^As their work shows, first-person accounts can reveal ideals and practices that remain invisible in more ‘formal’ sources such as handbooks and health manuals.^16^ Unfortunately, tracking down these accounts can be somewhat of a hit-and-miss exercise. Many diaries need to be checked to find a few useful accounts, and thus, I am glad that Friso Hoeneveld helped me with locating such accounts.

We started with two existing inventories of Dutch egodocuments.^17^ To locate medical memoirs, we used the inventory of published egodocuments, which contains almost 5,000 texts covering the long nineteenth century. Texts are included if their author is born after 1801 and at least part of the text covers a period within the time span 1800–1914. This means that, for example, texts starting in 1910 that also cover the 1920s and 1930s are also included. The texts have been categorised by author’s occupation. To locate medical memoirs, we extracted the texts with authors listed as having an occupation in the ‘medical sector’. This yielded 169 texts by 111 different authors. We further narrowed down our selection according to date (selecting only texts (potentially) covering (part of) 1900–1940), location (selecting only texts focusing on the Netherlands) and medical specialisation (focusing on general physicians, obstetricians and midwives; leaving out, for example, texts by pharmacists). Then, we selected based on actual content, through reading the texts to see whether they contained birth descriptions. This left 11 memoirs containing medical professionals’ accounts of childbirth, which I have studied closely for this article.

The memoir authors discussed their personal lives only sparingly, as is illustrated by the fact that only three authors included a chapter on their youth. The focus is on stories from their medical practice, which are told sometimes in chronological, sometimes in thematic order. The only exception is Aletta Jacobs, who also discussed her political activities: Jacobs was a well-known feminist. Some authors claim they tell these stories mainly to entertain; others express the hope that they will also educate their audience, by showing the progress medicine has made during their lifetimes.

To find sources from the parents’ perspective, we used the inventory of unpublished egodocuments and we visited archives which we knew had significant collections of first-person sources. Based on source descriptions in the egodocuments inventory and archive inventories, we selected sources that seemed likely to contain birth accounts (e.g., because the description explicitly mentioned a birth or because we knew from biographical details that the author had children). This resulted in 10 diaries and recollections with childbirth experiences, which I scrutinised for clues on the roles of fathers in childbirth.

I have found the memoirs and diaries to be rich sources, but, like all sources, they have limitations. The most obvious one is that they describe only doctor-led births. All the medical memoirs we have found were written by doctors. Both general physicians and specialised obstetricians are represented, but midwives are not. This is no coincidence. Historian Marijke Huisman has shown that in 1910, just over 25 per cent of autobiographies on the Dutch book market were written by women, and most of these were translations, leaving little space for memoirs authored by Dutch women.^18^ Such quantitative research has not been done for later decades, but it seems unlikely that anything changed soon after. In the introduction to her 1924 memoirs, female doctor Aletta Jacobs stated the lack of woman-authored memoirs in the Netherlands as a reason to publish hers.^19^ In addition to being female, midwives also came from lower classes than doctors did, again making them less likely to author memoirs.

I have looked specifically for midwife-authored memoirs outside the repository of egodocuments, through searching library catalogues and historical newspapers. This led to two potential candidates: *Als de ooievaar komt* and *Als het kindje komt*.^20^ Upon further inspection, both dropped out. *Als de ooievaar* komt, by Lisbeth Burger, is a translation from German, and thus describes German births, not Dutch ones. *Als het kindje komt* is presented as if written by a Dutch midwife, but I strongly suspect it to be fiction instead of life writing. I have not been able to find any sources indicating that the author, Martha Brouwer, indeed practised as a midwife (for all other memoirists, I could confirm their identities as doctors). Moreover, Martha Brouwer is a known pen name of the Dutch fiction writer Erna Koning-Manuel. In addition, the book differs from the other memoirs in form: the story is more dramatised, patient names are provided throughout the book (other authors usually anonymise patients), and the introductory remarks common in other memoirs are lacking. For these reasons, I have excluded the book from my analysis as not being an actual memoir.

The parents’ diaries also describe only doctor-led births, because the diarists come from higher classes, where families preferred doctors above midwives.^21^ This does not mean all doctor-led births took place in higher-class families; as we will see, fathers of all classes are represented in the sources.

For my argument that we need to study doctor-led births to understand the slow rise of hospital birth in the Netherlands, the absence of midwife-led births poses no problem. But for my argument on fathers’ presence, it does provide a challenge. In the public debate, doctors had a louder voice than midwives (as is illustrated by the lack of midwife memoirs), and in that sense, they may have had more influence on shaping cultural ideals on childbirth. But it remains possible that fathers were not, or less often, present at midwife-led births. Yet, I have seen some sources on midwife-led births: case reports in the midwifery journal *Tijdschrift voor Praktische Verloskunde* (*Journal for Practical Obstetrics*). They give no reason to suspect fathers attended less often when midwives supervised. A comprehensive study of the reports fell beyond the scope of this research, because they offer different birth descriptions than memoirs and diaries: more factual, less focused on feelings.^22^ This is due not to their authors, but to their form, being case reports intended to transmit medical information instead of personal stories. I have, however, carried out spot checks of the reports. Often, whether fathers attended the births remains unclear, but several reports that do mention them show that fathers certainly could attend midwife-led births.^23^

A second source characteristic to consider is that memoirs do not provide unmediated access to the historical truth. The time that historians understood memoirs as highly reliable accounts of what actually happened has long gone.^24^ Like all historical sources, memoirs are shaped by the conventions of their genre and the personality, intentions and beliefs of their authors. This makes them excellent sources to discover the values of their authors—such as their ideas on how fathers *should* behave during childbirth. But what about how fathers *did* behave during childbirth? Can the memoirs provide information on this as well? I think they can. We cannot assume stories in memoirs to be literally true, but we can assume that the memoirists believe them to be true. This distinguishes memoirs from novels: memoirists at least strive to describe the actual past.^25^ This justifies the assumption that there is at least some correspondence between the birth descriptions and the actual births, although the descriptions are subjective, and liable to the failings of human memory—the doctors wrote the memoirs towards the ends of their lives, decades after the events they described. Thus, we should not believe the doctors when they write that a certain birth took place on a Sunday afternoon or that the baby was the couple’s third daughter—it might just as well have been a Thursday morning, or the fourth son. But other, more general elements likely are more accurate, especially when confirmed by other sources, as is the case for fathers’ presence, which is confirmed by medical journals and parents’ diaries. Memoirists construct stories, but only stories that feel true to them. Thus, it is difficult to explain why doctors would regularly place fathers in the birthing room if we do not assume the fathers were actually there.

A last remark about the memoirs: they are written by doctors, but I use them partly to gain insight into the fathers’ experiences. Historians using, for example, case histories to study the patient’s perspective have demonstrated that sources written from the doctor’s perspective can be used in such ways, but it does require careful reading, since the fathers’ voices are mediated by the doctors.^26^ My use of parents’ diaries is helpful here, although these also not always provide direct access to the fathers’ voices, because sometimes it is the mother writing. Moreover, the memoirs remain crucial sources because they offer much more birth descriptions than the diaries do (doctors had more births to attend than parents) and because, unlike the diaries, they discuss also lower-class families.

I supplement the first-person sources with articles from medical periodicals, including the main obstetrics journal *Nederlandsch Tijdschrift voor Verloskunde en Gynaecologie* (*Dutch Journal for Obstetrics and Gynaecology*), the main general medical journal *Nederlandsch Tijdschrift voor Geneeskunde* (*Dutch Journal for Medicine*) and the *Tijdschrift voor Sociale Geneeskunde* (*Journal for Social Medicine*). To search for relevant articles, I used keyword search for the periodicals available digitally; for periodicals available in print only, I went through contents pages and indexes.

## The Ideal of the Harmonious Family

By the time the doctor arrived, the smoke had grown thick enough to slice it.^27^ The labour had already been going on for 48 hours, and the interested relatives, friends and neighbours attending it, had sat together in the room for two days and two nights—the men smoking, the women drinking brandy. The expectant father, a farmer, had fetched the doctor because the baby was stuck, with one arm hanging out of the mother’s body. The doctor, Aletta Jacobs, decided the situation required air and light and space: she opened the windows, had the brandy returned to the cellar and sent away most of the attendants. Some, however, could stay; the expectant father was one of them. The childbearing woman was transferred to the emptied table, and Jacobs set to work. By the end of the day, she had completed the delivery. The child had died, but, Jacobs wrote in her memoirs, ‘I tasted the satisfaction of having at least saved the woman.’^28^

Aletta Jacobs (1854–1929) published her memoirs in 1924, at the age of 70. By then she had acquired national fame as a prominent first-wave feminist. Jacobs was the first woman who was formally admitted to university and the first female doctor. She campaigned for birth control, against prostitution and, especially, for women’s right to vote. But the described birth took place long before these campaigns, in 1878, just a few months after Jacobs had passed the state exam for doctors. She had not yet established her own practice, but was covering for her father, a country doctor in Sappemeer, in the North of the Netherlands. Jacobs’ birth description has been cited previously by historical sociologist Rineke van Daalen, as an example of how, in the late nineteenth century, birth could still be a large social event, but modern medicine was starting to push out neighbours and relatives.^29^ Van Daalen thus focuses on who was sent away by Jacobs, and her sending away most attendants is of course significant. But here, I want to draw attention to the few people who were allowed to stay—and especially, to the fact that the expectant father was one of them.

In 1932, about half a century after Jacobs’ assistance at the 60-hour birth, obstetrician Maurits Lodewijk Muller (1884–1943) published an article on prolonged labour. Muller practised as a general physician for a few years, but worked in obstetrics for most of his career. He began his obstetrical training in 1909, in the city of Utrecht, where he also spent most of his working life, until he closed his obstetrical practice in 1940. He wrote down his work experiences in his memoirs, which he had almost finished when, in April 1943, the Nazis imprisoned him in camp Barneveld–Muller was Jewish. He died soon after, in September 1943. His memoirs were published posthumously in 1948. We will learn more about them later on; here I want to quote from his medical work, specifically the prolonged labour article. Muller stressed the importance of rest for all attendants of a prolonged labour. He advised his fellow obstetricians to administer morphine to the mother and organise a sleeping schedule for the others. If they were not ordered to bed, they would not go. Muller’s list of their reasons for staying awake provides us with an overview of persons present in the birthing room:

the nurse or *baker* [a type of maternity nurse] will not suggest it because she does not want to be considered hard-hearted, the expectant father considers it a moral duty to stay at the bedside of his spouse and all too often the anxious mother or mother-in-law keeps watch faithfully and in her state of agitation, she is frequently the biggest cause of the tense atmosphere.^30^

The first attendants mentioned by Muller are the nurse and the *baker*. One of these would usually assist the midwife or doctor during the birth. In the decades separating Jacobs and Muller, these nurses and *bakers* increasingly had received formal training and certification, although assistance from informally trained female neighbours also still occurred. The other two attendants on Muller’s list are relatives of the childbearing woman: the mother, or mother-in-law, and the expectant father. When we compare these two relatives with the smoking and brandy-drinking crowd encountered by Jacobs, it seems that the transition mentioned by Van Daalen—modern medicine pushing out neighbours and relatives—had continued. This is a development we know from other countries.^31^ But, as medical historian Anna Niiranen has shown for nineteenth-century Britain, the disappearance of large groups of lay attendants does not mean that medical professionals did not accept or value lay attendants at all.^32^ And indeed, Muller’s list may be short, but it does have space for lay attendants, including the father, who, according to Muller, considered sitting with his wife ‘a moral duty’.

Both Jacobs and Muller describe home births. In 1878, when Jacobs’ birth took place, virtually all births took place at home. In 1932, when Muller wrote his article, home births still formed the vast majority: according to government reports, 91.3 per cent of all births took place at home that year.^33^ But by then the number of hospital births had been rising for about 10 years. Dutch medical professionals had noticed the increase, and experienced it as rapid. In 1926, for example, the national health inspector wrote in his annual report that ‘the number of hospital births increased greatly’.^34^ Around the same time, several doctors published articles expressing concern about the growth of hospital births. These articles can help us understand why (some) fathers considered sitting with their labouring wives ‘a moral duty’. The doctors advocating against hospital birth stressed that birth was a family event that husband and wife should experience together. This view did not come out of nowhere, but dovetailed with broader ideas on family life within Dutch society, particularly the ideal of the harmonious family. Below, I will first outline this ideal, and then show how doctors arguing against hospital birth appealed to it.

In the first half of the twentieth century (as in many other eras), the family was widely seen as the cornerstone of Dutch society. I use ‘family’ here to translate *gezin*, which refers to the nuclear family of father, mother and children, and excludes extended family members such as grandparents, uncles, aunts and cousins. The Dutch had long been familiar with the nuclear family, both as an ideal and as an actual way of cohabitation. In the sixteenth and seventeenth century already—earlier than in other European countries—the combination father, mother, children was a common household form; having extended family members and household staff living in was less common than elsewhere.^35^ Van Daalen has argued that the seventeenth-century focus on the nuclear family was accompanied by a more privatised form of birth and childbed than before (and elsewhere), at least among some social groups.^36^ Family life was idealised; the Dutch housewife and Dutch domesticity became national and international tropes.^37^

In the following centuries, the ideal of the family would permeate Dutch society; its impact continues to be visible until the present day. From the middle of the nineteenth century, the Catholic and Protestant Churches intensified their efforts to propagate family life, as did private higher-class citizens wanting to ‘civilise’ the lower classes.^38^ From 1900, the state also became more involved. Interventions varied from the establishment of schools for knitting and sewing (which would later develop into domestic science schools), to an (unsuccessful) campaign for legislation prohibiting married women to have jobs, to architectural nudges in working-class homes, which were built with high-placed windows (to discourage participation in street life and help the residents focus on their own household) and small kitchens (to drive the family together in the living room).^39^

These interventions aimed to stimulate the ‘harmonious family’. ‘Harmonious’ was the standard adjective to describe the ideal family.^40^ In a harmonious family, each member had a specific role to play: the husband had to provide for his family; the wife had to take care of the home and the household; and the children had to be nice and obedient. In addition to properly playing their parts, all family members were supposed to spend lots of time together, mostly in their own homes. *Huiselijkheid* and *gezelligheid* were often presented as important values. *Huiselijkheid* translates as domesticity; *gezelligheid (*adjective *gezellig*) is a hard-to-translate word that can be applied to places, peoples and situations (from parties to canal trips to childbirths) that are cosy, homely, domestic and pleasant to experience.

Following the ideal of the harmonious family, men spent much of their leisure time at home, with their family, something contemporary British observer David Meldrum found remarkable.^41^ Meldrum also noted that, perhaps because of this, ‘the interests, the worries and the successes’ of Dutch men’s workaday life were ‘shared by the whole household more intimately than among ourselves’.^42^ It was indeed considered important for husband and wife to share life’s joys and sorrows.^43^

The ideal of the harmonious family was widespread: it transcended class, religion and political ideology. The higher classes may have believed the working classes needed to be ‘civilised’ before they could appreciate the pleasures of family life, but this reflected their own prejudices more than reality.^44^ Protestant and Catholic interpretations of the harmonious family may have differed subtly, but the similarities far outweighed these differences.^45^ Within politics, it was not just the confessional parties who considered the family the cornerstone of society; socialists and liberals were equally convinced of the importance of stimulating family life.^46^ The harmonious family was everywhere, and, as we will see now, doctors appealed to it when arguing against hospital birth.

## Birth as a Family Event—For All Classes

Obstetrician Andreas Wilhelmus Ausems (1871–1946) was one of the authors addressing the increase in hospital births in the 1920s. Like Muller, Ausems practised in Utrecht, where he also received his obstetrical training, in the early 1900s. Afterwards, he opened his own practice; he retired in 1942. During his career, Ausems published several books aimed at a general audience to spread his views on (social) matters related to obstetrics, which were shaped by his Catholic faith. Through his books and his participation in public debates, he gained national fame. His memoirs, published in 1938, would be reprinted at least five times.

In 1927, Ausems wrote a two-page article on hospital birth in the monthly journal for Catholic midwives that he edited. The article supplemented a shorter piece by midwife P. A. Jannes, a regular contributor to the journal. Jannes had read about plans to establish a Catholic birthing clinic for ‘normal’ births in Arnhem, a city in the East of the Netherlands, close to Nijmegen, where Jannes practised. Jannes disapproved of the plans, because she believed ‘the catholic idea is, that a normal birth should take place at home, because it is a healthy family event’.^47^ Ausems endorsed Jannes’ viewpoint with an extensive argument about (Catholic) birthing clinics not just in Arnhem, but in general, in which he also addressed the role of the husband.^48^

According to Ausems, moving normal births out of the ‘marital home’ was undesirable because it caused the loss of ‘a part of intimate married life’. Part of this intimate married life was the presence of the husband:

The presence of the husband during the delivery, the moral support and strength, which so many young mothers derive from this presence, an important factor in a harmonious marriage, is something that is eliminated in hospital care. Conversely, it is also absolutely undesirable, that the husband loses his share of the concern and anxiety during the delivery; that this concern is taken away by the suggestive white coats and the calming environment of the hospital delivery room. This share in the concern and anxiety is, after all, a new source of reviving love for his wife, who, for her part, needs the loving appreciation of her husband during all the pains she alone has to bear—the husband, for whom she endures all this.^49^

Note Ausems’ use of the phrase ‘harmonious marriage’; a direct reference to the ideal of the harmonious family. According to Ausems, the husband should attend the delivery not just to support his wife bearing the pain of the contractions, but also to fully experience the birth himself, including all the worrying that came with it. Husband and wife had to share the experience emotionally, to strengthen their marriage. This fits with the idea that, in a harmonious family, spouses shared life’s joys and sorrows with each other.

Jannes and Ausems wrote for a Catholic audience, but birth as a family event was not just a ‘catholic idea’, as Jannes put it. As mentioned above, the ideal of the harmonious family transcended religion; non-Catholic medical professionals were shaped by it just as well. Indeed, Ausems’ and Jannes’ pieces also resonated outside Catholic circles. The wide reach of their views is evinced by the circulation of their pieces. They were reprinted several times, in various forms. In Catholic magazines aimed at a broad audience, but also in two general medical journals aimed at all societal groups: the *Orgaan van den Bond van Nederlandsche vroedvrouwen (Organ of the Association of Dutch Midwives*) and *Nederlandsch Tijdschrift voor Geneeskunde* (*Dutch Journal for Medicine*; NTvG).^50^ The *Orgaan* took over both Jannes’ and Ausems’ pieces integrally. The NTvG mentioned both authors, but shortened the pieces considerably. Tellingly, however, the part about the husbands’ presence quoted above was copied almost in full, although the parenthesis ‘an important factor in a harmonious marriage’ was left out. Like the *Orgaan*, the NTvG was not aimed at a particular societal group. The journal stressed that, although Ausems had written about the deliveries of Catholic mothers, his words applied to all other mothers ‘certainly no less’.^51^

Non-Catholic doctors also wrote about birth as a family event elsewhere. In the same year as Ausems and Jannes, doctor C. J. Brenkman (1881–1951) discussed the rise in hospital births in the *Tijdschrift voor Sociale Geneeskunde* (*Journal for Social Medicine*). Between 1913 and 1919, he practised as a GP; in 1919, he started working for the medical services in Amsterdam. He became an active member of the non-denominational national association for social medicine and chaired the non-denominational health organisation Witte Kruis. After his death in 1951 (which his family announced in a liberal newspaper), a—laudatory—obituary in the Catholic medical journal *Katholieke Gezondheidszorg* (*Catholic Healthcare*) called him ‘not one of us’, meaning that Brenkman was not a Catholic. But he did consider birth to be a family event. He warned against the rise of hospital births, which he felt would weaken the position of midwives (who, not being allowed to supervise hospital births, lost part of their income). He did not mention the husband explicitly, but he is crystal clear on where to give birth: ‘A birth, if at all possible, needs to take place at home, being something that has nothing to do with illness, but that is an integral, beautiful and happy part of marriage and the *huiselijken haard.*’^52^

*Huiselijken haard* is difficult to translate. Literally, it means ‘domestic hearth’, but it has a strong metaphorical meaning as well. The dictionary *Woordenboek der Nederlandsche Taal* described it as ‘the centre of domesticity, *gezelligheid* and hospitality’.^53^ Birth, according to Brenkman, was a crucial part of marriage and domestic life. It belonged at home, in the sphere of domesticity and *gezelligheid*, both important values in the ideal of the harmonious family.

The same issue of *Tijdschrift voor Sociale Geneeskunde* that Brenkman published in also contained an article by doctor Claudius Henricus van Herwerden (1866–1962), who addressed the husband’s role. Van Herwerden, a former GP who had become director of the medical services in Rotterdam in 1919, came from a Protestant family. His father, a classics professor at Utrecht University, was born in the parsonage of Beetsterzwaag, where his grandfather was the Reformed pastor.^54^ As with Brenkman, his denomination differed from Ausems’, but his views on childbirth were similar.

Van Herwerden regretted the increase in hospital births because, as Brenkman also had argued, it threatened the position of midwives and, more relevant here, because it changed the atmosphere of the birth and diminished the role of the father. When higher-class women gave birth in the hospital, not much harm was done:

Although the domesticity and the special cachet that accompany the appearance of the little newcomer disappears with it [a hospital birth], the husband is present, and the childbearing woman has her own room, and thus, one may defend oneself with the assertion that the ‘home’ has been temporarily moved to the rented hospital room.^55^ [In the original Van Herwerden also uses the English ‘home.’]

But poorer women, who could not afford first-class treatment in private rooms, lost something crucial when they chose a hospital birth. They were admitted to maternity wards, surrounded by other women and without their husbands allowed to be present, making it impossible for the birth to be a family event. Van Herwerden:

Domesticity disappears completely; the endearing event that we all know and in which the husband participates fully, has changed into a hospital operation which fully or almost fully excludes him. The child is delivered to him ready to hand and he does not need to have the wind up at all. Moving the delivery to the public institution does away with all intimacy and undermines family life.^56^

Just as Brenkman, Van Herwerden refers to ‘domesticity’, an important value for the harmonious family. But even more important for Van Herwerden was the presence of the husband: he considers hospital birth acceptable for higher-class women even though domesticity disappears, because they can be together with their husbands, and thus are in a ‘home’ in the metaphorical sense. Poorer women, who cannot be with their husbands in the hospital, should avoid hospital birth because it ‘undermines family life’. At home, birth can be an ‘endearing event’ in which ‘the husband participates fully’. Note that this participation involves worrying—similar to Ausems, Van Herwerden considers it problematic that a hospital birth takes away a husband’s concerns during birth.

Van Herwerden stressed the importance of the husband’s presence for both higher- and lower-class women. This fits with the fact that, as mentioned earlier, the Dutch ideal of the harmonious family was seen as applying to all classes. But it is remarkable given the international historiography on fathers and childbirth. In her book *Family Men,* on fatherhood and masculinity in Britain, Laura King argues that between 1914 and 1960, the presence of men at childbirth was ‘extremely rare’ and that, if it happened, it was the fathers in ‘the most wealthy and upper-class families’.^57^ Only around 1960 did it become an acceptable option for other men as well. In nineteenth-century Britain, the practice had been somewhat more widespread, but research so far has focused mainly on the higher classes.^58^ In the early twentieth-century Netherlands, it appears to have been not rare at all for lower-class fathers to attend births. This appears not only from Van Herwerden’s article, but also from doctors’ memoirs.

Take, for example, the memoirs of obstetrician Muller. In the late 1910s, Muller worked as an assistant at the Utrecht university obstetrics clinic. The clinic provided care for poor patients, both in the hospital and at home, as part of their outpatient activities. Thus, Muller regularly biked to the small alleys in the Utrecht slums, where the houses were cramped and the supplies limited, but, according to Muller, the neighbours were always ready to help and the humour was good, ‘although, sometimes, rough’.^59^ The drunk accordionist we met above probably lived there. When Muller describes how many of the houses were crawling with vermin, he writes: ‘Fleas were common there and the many dark spots on sheets and pillow cases, if those were available, indicated their presence … Once, a jolly father-to-be pointed towards a large specimen while stating: “Look doctor, the stork has already arrived.”’^60^ The joke would work only if the expected baby had not come out yet—and since Muller praises the inhabitants of the slums for their sense of humour, we can assume the birth was indeed still ongoing. But to point out fleas to Muller, the father would have to be present at the birth.

In the memoirs of doctor L. van der Hoeven, who practised in the first decades of the twentieth century, we read about a baby girl being born at the Voldersgracht in The Hague.^61^ ‘Probably not at her own request’, Van der Hoeven added—the Voldersgracht had a particularly bad reputation, even for a slum.^62^ Two older children slept in the same room where the mother gave birth. Van der Hoeven: ‘When the new arrival started using its lungs, a little brother popped his head out of the box bed and asked: “Father, are there chickens in the house?” What a boy, the father said, proud of his oldest son.’^63^ As newborns start using their lungs immediately after birth, the father, who immediately commented on his son’s question, was in the room when the baby arrived.

Note that both Muller and Van der Hoeven mention the father as if his presence is self-evident. We will also see this in other birth descriptions from doctors’ memoirs, to be discussed in the next section. Doctors do not seem to feel the need to explain fathers’ presence in the birthing room, which shows how widely accepted this presence was.

However, not all fathers were present. Sometimes they were refused entrance, as we will see below. And sometimes they were unable or unwilling to attend. Being present was not a universal norm: in the memoirs of doctor Van der Heijden, we read that in his countryside practice, fathers only entered the birthing room after the baby had been born.^64^ And in his book on twentieth-century Dutch domestic life, historian Pieter Stokvis mentions that one of his respondents (Stokvis based his work on written questionnaires and interviews), a woman born in 1899, considered births ‘something for women’ and that it amazed her that her sons-in-law attended the birth of their children.^65^ This suggests that in her family, men did usually not attend births. But, my sources show, in many other families, they did: fathers’ presence may not have been universal, but it certainly was widespread, with doctors accepting or even encouraging it.

Thus, even though not all Dutch fathers attended the births of their children, we may conclude that the Netherlands differed from other countries, where, in the early twentieth century, fathers’ presence was highly unusual, and tended to be disapproved of by medical professionals. The Dutch ideal of the harmonious family can explain this difference. Birth was a family event, to take place at home, as an experience in which, as Van Herwerden put it, ‘the husband participates fully’. This participation required emotional involvement. In the next section, I will look at doctors’ memoirs and parents’ diaries to get an idea of how this emotional involvement took shape and of what ‘full participation’ may have looked—and felt—like in practice.

## Fathers’ Emotions

In the previous section, we saw how both Ausems and Van Herwerden considered it important that men felt at least somewhat concerned during childbirth; according to Ausems, this strengthened the marriage bond. The memoirs suggest that worrying indeed was a common emotion for expectant fathers—doctors often describe them with the adjectives *nerveus* and *zenuwachtig,* which both translate as ‘nervous’. As Ausems put it: ‘We observe a certain degree of nervousness in by far the most fathers, which is not surprising and hence one can hardly blame them.’^66^ Several authors illustrate this worrying with anecdotes about how fathers handled the night-bell when fetching the doctor. Getting the doctor (or the midwife) was a common task for expectant fathers, who, in addition to being emotionally involved, also regularly provided practical assistance during childbirth, with tasks that ranged from getting the doctor and holding lamps or legs to taking care of older children. Before the advent of the telephone, getting the doctor meant travelling to the doctor’s home and, if it was night-time, ringing the night-bell to wake him up. Nervous fathers tended to ring more than once. From the memoirs of obstetrician Muller: ‘At night, each second feels like a minute, and each minute, a century, for the expectant father who is on tenterhooks. Hence, there the finger goes again, towards the bouncy black button.’^67^ Ausems writes about opening the door to an expectant father after hearing the bell ring ‘long and shrilly’.^68^ And in the memoirs of doctor Van der Hoeven, we meet a father who was so nervous that the night-bell did not survive:

It even happened to me once, that a nervous expectant father pulled the bell out of the door. He came to get my help because his own doctor lived a few streets further, just five minutes by foot. Since I knew this colleague to be quite quick-tempered, I suggested to him to ring the bell a bit more discreetly, even more so because he told me that another colleague had already thrown him out for this reason.^69^

In these anecdotes, it is not just the adjective ‘nervous’ which suggests that the expectant fathers worried, but also the actions described by the memoirists. Ringing the bell loudly or repeatedly suggests a sense of urgency, more compatible with a nervous than a calm state of mind. The father described by Van der Hoeven in addition felt as if he lacked the time to get his own doctor, even though this doctor lived only a few streets further than Van der Hoeven. He also had been warned once already about his handling of the night-bell (by a doctor presumably living even closer to him than Van der Hoeven), and yet still was unable to ring Van der Hoeven’s bell carefully.

Some fathers seemed to manage joyful anticipation instead of, or in addition to, nervousness. Van der Hoeven again:

On a certain night someone was ringing the bell, on and on, to the tune of ‘I have carried my aunt to her grave’, which Cocquelin recited so wittily in those days. When I came downstairs, annoyed, I could not lash out, because it was a good friend who had come to get me for the first happy occasion in his family.^70^

Van der Hoeven goes on to describe the father as ‘cheerful’, which seems an appropriate description for someone managing to play well-known witty tunes on a night-bell.

Anticipatory joy could start long before the first contractions arrived. The diary of Albert Willem Wichers Hoeth (1876–1957) illustrates this. Wichers Hoeth, son of a judge, was a successful businessman. He became a partner in the firm Van Heekeren & Co and sat on several boards of trading associations. Between 1937 and 1941, he was a member of the Amsterdam Chamber of Commerce and, as such, also a governor at the Rotterdam School of Economics. In 1910, Wichers Hoeth married Henriëtta Maria Kuhn; soon after, she became pregnant. In the weeks before her due date, Wichers Hoeth regularly refers to the upcoming birth and the accompanying preparations: ‘Lo and behold the pram, the crib, the stacks of postcards already written.’^71^ He speculates about the sex of the expected baby—a girl, he thinks. On 31 May 1911, their first wedding anniversary, he writes: ‘Our annual report can only report happiness and more happiness; today still for the two of us, tomorrow perhaps for the three of us.’^72^ His predictions turned out to be correct, because on 1 June his wife gave birth to a daughter. Labour had started at the evening of their wedding anniversary, we read in the diary: ‘It has been a trying day, after a night without sleep, because the prologue had already started last night, and therefore I am also going to bed now.’^73^ Note that Wichers Hoeth states that he did not sleep, which strongly suggests he sat with his wife all night—if he did not accompany her, why would he not have had at least a few hours of sleep? But although the birth had been tiring, Van der Hoeven’s writing radiates joy:

Haven’t I always said, haven’t I trumpeted to everyone willing to listen, that it would be a girl? She, our Henriette Cornelia, appeared around 3.30, and how! Really mettlesome! Of course, a delivery doesn’t go without pains, but it was beautiful, how brave my Jetje [Henriëtta] was, she didn’t make a sound, so that Anne, the maid, stated to me later that she thought that the whole family had fallen asleep.^74^

Wichers Hoeth is proud of his wife, and tremendously happy with his new daughter. He enjoyed the hours after the birth, which he shared with his wife: ‘Oh! How wonderfully cheerful Jet was again, once she had lost her parcel, and how pleasantly did I sit at her bedside all evening, as if she had experienced a small headache.’^75^

In the memoirs, we also find happy fathers: after the child arrived, nervousness was often replaced with joy, relief and gratitude. Muller reflects on women who receive gifts from their husbands after giving birth, which he explains to be expressions of gratitude to the wife: ‘She has, after all, given him his son, whose first cry he greeted with a sob, and she has felt his trembling mouth on hers when he threw himself around her neck, stating “Thank goodness, it has arrived.”’

But not all fathers felt relieved after a birth had taken place. Births do not always end well. Three years after Wichers Hoeth became a father for the first time, Marxist politician Willem van Ravesteyn (1869–1970) experienced what he would later, at the age of 87, describe as ‘perhaps the largest catastrophe of my life’.^76^ In the summer of 1914, Van Ravesteyn and his wife Johanna Wismeijer were expecting their first child; by then, they had been married for almost four years. In the middle of a late August night, Wismeijer felt the first contractions. Their doctor came, and only then established that Wismeijer’s pelvis was too small for a vaginal birth—but by then, it was too late for a c-section. The doctor called in the prominent obstetrician Klaas de Snoo, director of the Rotterdam Midwifery School and author of the first Dutch handbook of obstetrics.^77^ Transport to the hospital was unavailable, so De Snoo had to help Wismeijer at home. To save the mother’s life, he had to make the unborn child, a girl, fit through the pelvis—which meant crushing the child’s skull. And Van Ravesteyn had to assist him. From the recollections he wrote down for his son:

So the man had to—at home, with just me as an assistant (for handing a basin and so on)—proceed to kill the little creature that had to be born, i.e., shatter the little head in order to save the mother. Was this last thing the case? I don’t know. Anyhow: it happened and what’s more, I had to assist in this horrible and bloody incident: murder on a young female creature. Once again: I don’t recall any details, fortunately. The child was a girl. … And this has, in my mind, made things even worse, because I had been hoping for a little girl.^78^

Note that the severe complications did not mean that the husband had to leave the room. Quite the opposite: his practical assistance was required. Van Ravesteyn does not recall what happened in the hours after the dead girl was born. But, he continues:

one does not need much imagination to call to mind the horrible character of the situation. After dr. De Snoo had left, I must have remained behind, on my own with a badly wounded wife, a bleeding child’s corpse, and nothing else. A situation which perhaps can be compared with the horrors of war that had become usual in Europe since the first of August.^79^

Van Ravesteyn’s inability to describe what he felt after the birth suggests that the event was so traumatic that he partly suppressed the memories of it. The whole ordeal made a life-long impression on him. He explicitly states this in his recollections; it also appears from his diary.^80^ Decades later, on 26 August 1955, he wrote: ‘Yesterday and today, I am again thinking of the catastrophe of 41 years ago. 41 years old she would have been now, the murdered little one.’^81^

Most men had less traumatic birth experiences than Van Ravesteyn. However, even a birth ending with mother and child alive and well, did not always make fathers happy. Ausems wrote in his memoirs how fathers can be ‘grumblers who immediately start complaining to the obstetrician about the bad times and the worries that children cause’.^82^ In the memoirs of general physician Coster we read about a father who was ‘a crabby bloke, perhaps also due to the fact that the spouses expected their 13th child’.^83^ After the *baker* joked about this, the husband responded: ‘If you’d care to have it, sir, I’d be happy to give it to you for free!’^84^ Coster reacted by taking the man to an adjacent room and telling him off, stating that he should be ashamed ‘saying such things in the presence of your wife who has endured so much pain’.

Some fathers may not have welcomed their children, but Betsy Tierolff’s husband certainly did. Betsy Tierolff (1906–98) was born in Roosendaal, in the South of the Netherlands, in 1906. She came from a wealthy family: in her diary, she describes her younger self as ‘belonging to the rich children’.^85^ In 1925, she became engaged with Jan Nagel, a school teacher, who then earned 280 guilders a week, enough to reassure her parents that Jan would be able to provide financial stability.^86^ Jan and Betsy settled in the city of Nijmegen, where they lived in a house at the Berg en Dalscheweg. Half a century after they moved in, Betsy wrote down some memories attached to the house, where she still lived, on her own now, because Jan had passed away. One room especially evoked strong feelings, ‘our large, delightful bedroom with balcony’, where she gave birth four times, in the 1930s and 1940s. She described the births as joyful occasions:

Four of our six children were born in this room and what an experience it was each time! Father each time so impressed that he could barely hold back his tears. How welcome each child was and how happy we were with them; we experienced unforgettable moments when, after the delivery, we examined our ‘new child’ together.^87^

Betsy describes Jan as ‘impressed’ and as emotionally involved in the birth. They share the experience; they are in the room together. Betsy uses the pronoun ‘we’, suggesting togetherness. They share similar emotions: both of them are ‘happy’, and in admiring the new baby together, they share an ‘unforgettable moment’. Jan could ‘barely hold back his tears’, which not only suggests emotional involvement, but also that he tried to control his emotions. He was on the verge of crying, but he did not cry. Instead, he held back his tears, albeit barely.

In 1909, a long-awaited baby arrived in the grandest home of the country: the royal palace in The Hague. And here, we also see the father both displaying and controlling his emotions. Queen Wilhelmina and Prince Hendrik had been waiting for an heir for over seven years. Wilhelmina lost three pregnancies, in 1901, 1902 and 1906. During the second pregnancy, she became very ill herself.^88^ In 1908, a fourth pregnancy came, and stayed. The baby was due in April 1909, and on the 28th of that month, Wilhelmina’s labour began. After two days, the baby arrived.

The long labour and troublesome history provided Hendrik with ample time and reason to worry. Indeed, newspaper correspondents described him as nervous. Newspaper *De Courant* wrote during the early stages of labour that Hendrik ‘appears to be very nervous. In the morning, he paced up and down the front garden of the palace with hurried steps for a good length of time’.^89^ As Laura King has pointed out, husbands’ attendance did not necessarily mean continuous presence but could take many forms, especially during home births.^90^ During long labours such as Wilhelmina’s, it seems almost inevitable that husbands stepped out every now and then, if only to stretch their legs or to get some rest. A nurse present at Wilhelmina’s birth described in a letter to her family how, on the first day, she saw Hendrik and Wilhelmina sitting together, having lunch, when she entered the room.^91^ Hendrik then stepped out when the nurse questioned the queen, and entered again when she left. During the final stages of labour in the early morning of 30 April, Hendrik left the room to lay down on a camp bed close to the labour room to rest. According to national newspaper *De Telegraaf,* he was ‘overcome by the emotions of the past few days’.^92^ Hendrik’s resting period was short: he was called back into the labour room after an hour, at 6 AM, because the actual birth was near. Just before 7 AM, the little princess was born. The news was first announced by a servant, then confirmed by the queen’s private secretary, and then, the prince himself came out:

With tears in his eyes, he runs downstairs and simply shouts: ‘a Princess’ … [ellipses in the original] This resounded through the old, large, quiet palace, from downstairs to upstairs; then, suddenly, he becomes His Royal Highness again. In a stiff military fashion, he accepts the first congratulations, offered by Her Majesty’s adjutants.^93^

In announcing the birth, Hendrik displayed emotions—he had tears in his eyes. But he then quickly regains control of his emotions and hides them, becoming ‘His Royal Highness again’. During the birth, Hendrik also experienced emotions, up to the point that he, according to *De Telegraaf*, was ‘overcome’ with them. Note that, when this happened, Hendrik left the room. Being overcome with emotions was apparently an indication that he needed rest, which suggests that once the expectant father was too emotional, he should not be in the birthing room.

Other birth descriptions also place fathers with uncontrolled emotions outside the birthing room. Muller’s memoirs offer an example. On a rainy night, Muller was called to assist at a slowly progressing labour by the local doctor. Muller and the doctor decided to use the forceps. Muller describes how the legs of the woman were held by the local doctor and a neighbour, and then continues: ‘The husband was in a complete muddle and stood sobbing in the back kitchen; he was useless to us.’^94^ The husband had lost control of his emotions: he was not just nervous, but ‘in a complete muddle’ and he did not have a few tears in his eyes, but ‘stood sobbing’. This made him ‘useless’; he was in no state to, for example, hold a leg, or to emotionally support his wife.

Muller’s writing nowhere suggests the husband was sent away; it seems that he left (or never entered) the birthing room of his own accord. But sometimes husbands were explicitly asked to leave; think, for example, about the accordionist who opened this article. Ausems addresses the removal of husbands in his memoirs:

[T]he obstetrician is usually not particularly keen on the father’s presence and when he [the father] in his turn is keen on being as close as possible to his wife, he generally sits highly inconveniently in the way, while not seldom in doing so pretending to be the one suffering, needing to be treated with empathy and consideration. Matters get even worse as he plays this part with greater conviction, and thus becomes bothersome and unpleasant not just for the obstetrician but also for his wife.

Then there is nothing for it but to ask him to, meanwhile, go somewhere else.^95^

First, note that however much Ausems may have stressed that fathers should participate in the birth, he did not always enjoy this participation in practice. He admits that fathers could annoy him and claims that his colleagues shared his irritation. Gerrit de Jong, a doctor in Amsterdam, was such a colleague. In response to the articles by Van Herwerden and Brenkman discussed above, De Jong wrote that, in his eyes, ‘such a nervous *kraamheer* [a man whose wife is giving birth or has just given birth] can easily be missed, but this is a matter of taste’.^96^ Yet, De Jong seems to have accepted fathers’ presence nonetheless. For Ausems, annoying behaviour in itself was no reason to send the father away either, as long as he did not bother the expectant mother.

That both De Jong and Ausems accepted the presence of fathers who annoyed them confirms once more how normal this presence apparently was. But there were limits. Ausems drew the line at behaviour that bothered not just the obstetrician but also the woman in labour. This happened when men played the part of ‘pretending to be the one suffering …. with greater conviction’. In what follows Ausems further specifies this part: it was fathers with ‘desperate and forlorn looks in their eyes’, of whom the worst ‘complain and groan along’ with each contraction, ‘as if [they] also endured the labour pains’.^97^ What Ausems describes here are expectant fathers who display strong emotions. They are not just nervous, but ‘desperate’; they appear to feel as if they suffer from the pains of labour themselves, and make accompanying noise. Ausems considers this an inappropriate way to display emotions, that did not belong in the birthing room.

Ausems implies that such an uncontrolled display of emotions transgressed gender norms, writing that a man who groans along with each contraction does not understand ‘that such an attitude is fatal to his dignity as a man’.^98^ Men apparently were not supposed to behave this way. But as pointed out above, Ausems’ disapproval also stemmed from the fact that the behaviour bothered the woman in labour.

That husbands should not overshadow their wives also follows from how Ausems and Muller dealt with fainting fathers. Some men were so affected by the physical and emotional aspects of the birth experience that they fainted. Ausems writes, for example, about a farmer from Kockengen, who had offered to lend a hand during a difficult labour. Ausems gratefully accepted the offer, but just when the farmer’s assistance was most needed, ‘he sank down solemnly, slowly and soundlessly’.^99^ The unconscious farmer was lying in the way, and thus Ausems used his feet to push him under the (raised) bed, after which he proceeded with the delivery. ‘Mother and child required quite some care, and hence, we completely forgot the young father.’^100^ Muller’s memoirs contain a similar anecdote, now about a policeman. He was holding a paraffin lamp to light the box bed in which Muller was delivering his wife, who had been put under narcosis because of complications. Suddenly, the man fainted and dropped the lamp, which almost set the house on fire. Luckily, one of the assisting neighbours could grab the lamp just in time, and the work continued. ‘For the time being, we could not concern ourselves about the unconscious husband, who was lying on the ground like a corkscrew and had been pulled into a corner by the arms of the ministering angel: first things first.’^101^

First things first: the mother came before the father. But, as should be clear by now, fathers had a place in the birthing room as well—even if they annoyed doctors, they were not necessarily send out. When present, fathers should be emotionally involved in the birth. My analysis of the memoirs and diaries suggests fathers experienced a range of emotions, but also revealed that not all displays of emotion were equally appropriate. In particular, fathers should remain in control of their emotions. Men who were overtaken by their emotions had no place in the birthing room.

## Conclusion

Not all fathers were present when their children arrived, but many were, and medical men supervising births usually accepted their presence—sometimes happily, sometimes grudgingly. This distinguishes the Netherlands from other countries, in particular the Anglo-Saxon ones. The difference can be explained through the ideal of the harmonious family, which permeated Dutch society in the first half of the twentieth century, and which dovetailed with men sharing the birth experience with their wives.

Dutch doctors considered birth a family event, and thus let fathers into the birthing room. In the birthing room, fathers were expected to be emotionally involved: they had to display emotions without losing control of them. My findings complicate existing historiography on fathers and childbirth. In addition, they illustrate how a doctor-led birth often was a family event, taking place at home. Getting a doctor to supervise a birth did not mean that family members had to leave the room or that the birth should move to the hospital, not even if the doctor was a specialised obstetrician. We have seen how obstetrician Muller supervised births in the houses of the poor; how fellow obstetrician Ausems even actively opposed hospital birth; and how both obstetricians, as well as most other doctors we have met, allowed husbands to share the birth experience with their wives, making birth, in the words of doctor Brenkman, ‘something that has nothing to do with illness, but that is an integral, beautiful and happy part of marriage and family life’.^102^

All this weakens the idea that we should solely focus on the midwife to explain the slow rise of hospital birth in the Netherlands. To fully understand the Dutch birthing system, and the history of home and hospital birth more general, we need to study doctors as well as midwives. It may have been Dutch doctors, just as much or perhaps even more than the Dutch midwives, who kept Dutch births in the family.

## Footnotes

1 Maurits Lodewijk Muller, *Aan de poort van het leven: Herinneringen van een vrouwenarts* (Utrecht: Bruna, 1948), 20. In the original quote, the husband talks in local dialect.

2 Two exceptions, which unfortunately discuss other time periods than this article: Magdalena S. Sánchez, ‘“I Would Not Feel the Pain If I Were with You”: Catalina Micaela and the Cycle of Pregnancy at the Court of Turin, 1585–1597’, *Social History of Medicine*, 2015, 28, 445–64; Ema Hrešanová, ‘The Island of Alternatives: Power, Medical Science, and “Gentle Birthing” in Socialist Czechoslovakia’, *Journal of the History of Medicine and Allied Sciences*, 2018, 73, 73–95.

3 Jill Suitor, ‘Husbands’ Participation in Childbirth: A Nineteenth-Century Phenomenon’, *Journal of Family History*, 6, 1981, 278–93.

4 Patricia Jalland, *Women, Marriage and Politics: 1860–1914* (paperback edition; Oxford: Oxford University Press, 1988), 144–49; Judith Schneid Lewis, *In the Family Way: Childbearing in the British Aristocracy, 1760–1860* (New Brunswick: Rutgers University Press, 1986), 170–73; see also John Tosh, *A Man’s Place: Masculinity and the Middle-Class Home in Victorian England* (New Haven: Yale University Press, 1999), 80–82.

5 Leanne Calvert, ‘“A More Careful Tender Nurse Cannot Be Than My Dear Husband”: Reassessing the Role of Men in Pregnancy and Childbirth in Ulster, 1780–1838’, *Journal of Family History*, 2017, 42, 22–36.

6 Laura King, *Family Men: Fatherhood and Masculinity in Britain, 1914–1960* (Oxford: Oxford University Press, 2015), 175–77; Laura King, ‘Hiding in the Pub to Cutting the Cord? Men’s Presence at Childbirth in Britain c. 1940s–2000s’, *Social History of Medicine*, 2017, 30, 389–407; Judith Walzer Leavitt, ‘What Do Men Have to Do with It? Fathers and Mid-Twentieth-Century Childbirth’, *Bulletin of the History of Medicine*, 2003, 77, 235–62; Judith Walzer Leavitt, *Make Room for Daddy: The Journey from Waiting Room to Birthing Room* (Chapel Hill: University of North Carolina Press, 2009); see also Suitor, ‘Husbands’ Participation’, 289.

7 Centraal Bureau voor de Statistiek, *1899–1979, Tachtig jaren statistiek in tijdreeksen* (The Hauge: Staatsuitgeverij, 1979), 32.

8 King, ‘Men’s Presence’, 391; Judith Walzer Leavitt, *Brought to Bed: Childbearing in America, 1750–1950. 30th Anniversary Edition* (Oxford: Oxford University Press, 2016), 171.

9 Statistics’ Netherlands Database Statline (table name ‘Bevalling en geboorte: 1989–2013’, accessed on 1 September 2022), https://opendata.cbs.nl/#/CBS/nl/dataset/37302/table?dl=6DF70.

10 Anja Hiddinga, ‘Verloskunde in Nederland: Vroedvrouwen en de thuisbevalling’, in Rineke van Daalen and Marijke Gijswijt-Hofstra, eds, *Gezond en wel: Vrouwen en de zorg voor gezondheid in de twintigste eeuw* (Amsterdam: Amsterdam University Press, 1998), 189–204, 189. For examples, see Eva Abraham-Van der Mark, ed., *Successful Home Birth and Midwifery: The Dutch Mode* (Amsterdam: Het Spinhuis, 1996), ix–xi; Wendy Christiaens, Marianne Nieuwenhuijze and Raymond De Vries, ‘Trends in the Medicalisation of Childbirth in Flanders and the Netherlands’, *Midwifery*, 2013, 29, e1–8, e5–6.

11 The figures on birth supervision mentioned here and in the remainder of this paragraph come from ‘Verslag van den hoofdinspecteur van de volksgezondheid […] 1924’, *Verslagen en mededeelingen betreffende de volksgezondheid*, 1925, 905–1012, 972–73; Centraal Bureau voor de Statistiek, *1899–1979*, 32.

12 Hilary Marland, ‘The Guardians of Normal Birth: The Debate on the Standard and Status of the Midwife in the Netherlands Around 1900’, in Abraham-Van der Mark, *Successful Home Birth*, 21–44, 29–33.

13 For hospital births in 1900, see: Rineke van Daalen, ‘De groei van de ziekenhuisbevalling: Nederland en het buitenland’, *Amsterdams Sociologisch Tijdschrift*, 1988, 15, 414–45, 417. From the 1920s onwards, figures on hospital births can be found in the reports of the public health inspector published in the yearly *Verslagen en mededeelingen betreffende de volksgezondheid*.

14 John R. Gillis, *A World of Their Own Making: A History of Myth and Ritual in Family Life* (Oxford: Oxford University Press, 1997), 191; Lewis, *In the Family Way*; Anna Niiranen, ‘“The Health and Happiness of the Expectant Mother”: Constructions of Pregnancy and Childbirth in British Medical Writings, 1840–1902’ (unpublished PhD thesis, University of Jyväskylä, 2020), 260.

15 Jalland, *Women, Marriage and Politics*; Leavitt, *Make Room for Daddy*; Lewis, *In the Family Way*; King, ‘Men’s Presence’.

16 On the relative absence of fathers in these sources, see Niiranen, ‘Health and Happiness’, 258.

17 The published egodocuments inventory can be found at http://resources.huygens.knaw.nl/egodocumenten; the unpublished egodocuments inventory, at www.egodocument.net/repertorium.html.

18 Marijke Huisman, *Publieke levens: Autobiografieën op de Nederlandse boekenmarkt 1850–1918* (Zutphen: Uitgeversmaatschappij Walburg Pers, 2008), 211–12.

19 Aletta Jacobs, *Herinneringen* (Amsterdam: Van Holkema, 1924), v.

20 Lisbeth Burger, *Als de ooievaar komt: Lief en leed uit de praktijk van een vroedvrouw* (Amsterdam: Blitz, 1933); Martha Brouwer, *Als het kindje komt...: Uit het dagboek van een Amsterdamse vroedvrouw* (Amsterdam: Scheltens & Giltay, 1937).

21 Andreas Wilhelmus Ausems, *Tienduizend baby’s: Herinneringen en overpeinzingen* (Utrecht: Spectrum, 1938), 164; C. Campagne-Busces, ‘Onzuivere mededinging in het vroedvrouwenberoep’, *Tijdschrift voor Praktische Verloskunde*, 1901, 5, 250–51.

22 Feelings are not always absent, however, see, for example, vol. 3 (1899–1900), 345–46.

23 For examples of fathers attending, see vol. 5 (1901–02), 58–60; vol. 20 (1915–16), 175; vol. 27 (1922–23), 426; vol. 42 (1938–39), 31–34.

24 On the evolution of memoirs as historical sources, see Michael Mascuch, Rudolf Dekker and Arianne Baggerman, ‘Egodocuments and History: A Short Account of the Longue Durée’, *The Historian*, 2016, 78, 11–56.

25 Paula S. Fass, ‘The Memoir Problem’, *Reviews in American History*, 2006, 34, 107–23, 121; G. Thomas Couser, *Memoir: An Introduction* (Oxford: Oxford University Press, 2011), 55.

26 See, for example, Nancy Schrom Dye, ‘Modern Obstetrics and Working-Class Women: The New York Midwifery Dispensary, 1890–1920’, *Journal of Social History*, 1987, 20, 549–64; Guenter B. Risse and John Harley Warner, ‘Reconstructing Clinical Activities: Patient Records in Medical History’, *Social History of Medicine*, 1992, 5, 183–205.

27 Jacobs, *Herinneringen*, 51–52.

28
*Ibid*., 52.

29 Rineke van Daalen, ‘Verloskundige oefeningen: Een student in Leiden, 1919’, in Van Daalen and Gijswijt-Hofstra, *Gezond en wel*, 205–12, 207.

30 Maurits Lodewijk Muller, ‘Langdurige baringen’, *Nederlandsch Tijdschrift voor Geneeskunde*, 1932, 76, 5733–44, 5742.

31 Leavitt, *Brought to Bed*.

32 Niiranen, ‘Health and Happiness’, 267–70.

33 ‘Verslag omtrent het toezicht […] 1932’, *Verslagen en Mededeelingen betreffende de Volksgezondheid*, 1934, 333–94, 391. This figure is probably slightly too high because births in the three training clinics attached to the four Dutch midwifery schools were not registered as hospital births.

34 ‘Verslag van de hoofdinspecteur […] 1926’, *Verslagen en Mededeelingen betreffende de Volksgezondheid*, 1928, 125–258, 250.

35 Dirk Damsma, *Familieband: Geschiedenis van het gezin in Nederland* (Utrecht: Kosmos, 1999), 46; Simon Schama, *The Embarrassment of Riches: An Interpretation of Dutch Culture in the Golden Age* (London: Fontana Press, 1991), 386–87.

36 Rineke van Daalen, ‘Family change and continuity in the Netherlands: Birth and childbed in text and art’, in Abraham-Van der Mark, *Successful Home Birth*, 77–94.

37 Dirk Damsma and Els Kloek, ‘‘T’Huys Best’ Huiselijkheid in Holland sedert de Gouden Eeuw’, *Holland*, 2012, 44, 100–109, 101–4; Els Kloek, *Vrouw des huizes: Een cultuurgeschiedenis van de Hollandse huisvrouw* (Amsterdam: Balans, 2009); A. J. Schuurman, ‘Is huiselijkheid typisch Nederlands? Over huiselijkheid en modernisering’, *BMGN*—*Low Countries Historical Review*, 1992, 107, 745–59; Schama, *The Embarrassment of Riches*, 384–400.

38 Damsma, *Familieband*, 112, 138.

39 Dirk Damsma, ‘Van hoeksteen tot fundament: Het gezin in Nederland 1850–1960’, in Harry Peeters, Lène Dresen-Coenders and Ton Brandenbarg, eds, *Vijf eeuwen gezinsleven: Liefde, huwelijk en opvoeding in Nederland* (Nijmegen: SUN, 1988), 209–47, 232; Damsma, *Familieband*, 143.

40 J. C. H. Blom, ‘Een harmonisch gezin en individuele ontplooiing: Enkele beschouwingen over veranderende opvattingen over de vrouw in Nederland sinds de jaren dertig’, *BMGN*—*Low Countries Historical Review*, 1993, 108, 28–50; Kloek, *Vrouw des huizes*, 180–81; Pieter Stokvis, *Het intieme burgerleven: Huishouden, huwelijk en gezin in de lange negentiende eeuw* (Amsterdam: Bert Bakker, 2005), 131–82.

41 D. S. Meldrum, *Home Life in Holland* (London: Methuen, 1911), 73, 77; see also: Kloek, *Vrouw des huizes*, 207.

42 Meldrum 1911, 77.

43 Stokvis, *Het intieme burgerleven*, 157; Damsma, ‘Gezin in Nederland’, 217.

44 Kloek, *Vrouw des huizes*, 180–81.

45 Damsma, ‘Gezin in Nederland’, 218.

46
*Ibid.*, 230–40; Damsma, *Familieband*, 113.

47 P. A. Jannes and Andreas Wilhelmus Ausems, ‘R. K. Kraamkliniek’, *Maandblad voor R. K. vroedvrouwen* 4, 1927, 168–71.

48
*Ibid.*

49
*Ibid.*, 169.

50 P. A. Jannes and Andreas Wilhelmus Ausems, ‘R. K. Kraamkliniek’, *Katholiek sociaal weekblad*, 1927, 26, 296–97; ‘R. K. Kraamkliniek’, *Mannenadel en vrouweneer*, 1927, 17, 14–16; P. A. Jannes and Andreas Wilhelmus Ausems, ‘R. K. Kraamkliniek’, *Orgaan van den Bond van Nederlandsche Vroedvrouwen*, 1927, 9, 52–53; ‘Bevalling thuis of in de cliniek’, *Nederlandsch Tijdschrift voor Geneeskunde*, 1927, 71, 323–24.

51 ‘Bevalling’, 324.

52 C. J. Brenkman, ‘De positie der vroedvrouw bedreigd’, *Tijdschrift voor Sociale Geneeskunde*, 1927, 5, 135–38, 138.

53
*Woordenboek der Nederlandsche Taal*, s.v. ‘haard’, entry published in 1897, consulted online at https://gtb.ivdnt.org

54 P. C. Molhuysen and Fr. K. H. Kossmann, eds, *Nieuw Nederlandsch Biografisch Woordenboek*, vol. 10 (Leiden: Sijthoff, 1937), s.v. ‘Herwerden, Henricus van’.

55 C. H. van Herwerden, ‘De trek naar de kraamzaal’, *Tijdschrift voor Sociale Geneeskunde*, 1927, 5, 134–35, 135.

56
*Ibid*.

57 King, *Family Men*, 175–77; quotations on page 175.

58 Jalland, *Women, Marriage and Politics*, 144–49; Lewis, *In the Family Way*, 170–73; Tosh, *A Man’s Place*, 80–82.

59 Muller, *Herinneringen*, 15, 18.

60
*Ibid*., 14.

61 L. van der Hoeven, *Uit de praktijk van een medicus* (The Hague: Mouton, 1929), 24.

62 Auke van der Woud, *Koninkrijk vol sloppen: Achterbuurten en vuil in de negentiende eeuw* (Amsterdam: Bert Bakker, 2010), 92, 117.

63 Van der Hoeven, *Uit de praktijk*, 24. In the original quotation, the boy talks in local dialect.

64 I. van der Heijden, *Een arts op zijn praatstoel* (Amsterdam: Strengholt, 1960), 28.

65 Pieter Stokvis, *Huishouden, huwelijk, gezin: Huiselijk leven in de 20e eeuw* (Rotterdam: Donker, 2003), 102.

66 Ausems, *Tienduizend baby’*s, 94.

67 Muller, *Herinneringen*, 11.

68 Ausems, *Tienduizend baby’*s, 46.

69 Van der Hoeven, *Uit de praktijk*, 59.

70
*Ibid*., 58–59.

71 Travel reports and memoirs of Albert Willem Wichers Hoeth, book IX, 1911–14, page 202, entry May 1911, file 173, Family papers Hoeth and Wichers Hoeth, 894, Groninger Archieven [Groningen Archives], Groningen.

72
*Ibid.*, 204, entry 31 May 1911.

73
*Ibid.*, 205, entry 1 June 1911.

74
*Ibid.*, 204, entry 1 June 1911.

75
*Ibid.*, 204–5, entry 1 June 1911.

76 Willem van Ravesteyn, ‘Zelfportret voor mijn zoon’, 1963–65, page 57, file 33, Willem van Ravesteyn papers, 53-02, Stadsarchief Rotterdam [Rotterdam Archives], Rotterdam.

77 Klaas de Snoo, *Beknopt leerboek der verloskunde* (Groningen: Wolters, 1910).

78 Van Ravesteyn, ‘Zelfportret’, 58.

79
*Ibid*.

80
*Ibid*.

81 Diary Willem van Ravesteyn, folder VI, 3/1/1955–27/12/1956, page 721, entry 26 August 1955, file 30, Willem van Ravesteyn papers, 53-02, Stadsarchief Rotterdam, Rotterdam.

82 Ausems, *Tienduizend baby’s*, 92.

83 P. P. Coster, *Huisdoktersleven* (Assen: Van Gorcum, 1956), 35.

84
*Ibid.*

85 Typescript of the recollections of Betsy Nagel-Tierolff, 1976–87/90, page 32, Collection egodocuments, EGO/188, Atria Archives, Amsterdam.

86
*Ibid.*, 40.

87
*Ibid.*, 68.

88 Science studies scholar Trudy Dehue has recently argued that Wilhelmina’s second pregnancy loss was an induced abortion carried out to save her life. Trudy Dehue *Ei, foetus, baby: Een nieuwe geschiedenis van zwangerschap* (Amsterdam: Atlas Contact, 2023), 176–83.

89 ‘De blijde verwachting’, *De Courant*, 29 April 1909.

90 King, ‘Hiding in the Pub’, 393.

91 Report on the birth of H.R.H. Princess Juliana by Maria Johanna van Persijn, 1909, page 5, file 342, Family papers Van Persijn, 1350-01, Haags Gemeentearchief [The Hague Archives], The Hague.

92 ‘De blijde gebeurtenis’, *De Telegraaf,* 30 April 1909, evening edition.

93
*Ibid.*

94 Muller, *Herinneringen*, 184.

95 Ausems, *Tienduizend baby’s*, 89.

96 G. J. de Jong, ‘De kraamzaal, de vroedvrouw en nog iets’, *Tijdschrift voor Sociale Geneeskunde*, 1927, 5, 154–55, 154.

97 Ausems, *Tienduizend baby’s*, 90.

98
*Ibid*.

99
*Ibid*., 91.

100
*Ibid*.

101 Muller, *Herinneringen*, 136.

102 Brenkman, ‘De positie der vroedvrouw bedreigd’, 138.

